# Effect of Nano-HA/Collagen Composite Hydrogels on Osteogenic Behavior of Mesenchymal Stromal Cells

**DOI:** 10.1007/s12015-016-9644-x

**Published:** 2016-01-23

**Authors:** Astghik Hayrapetyan, Matilde Bongio, Sander C. G. Leeuwenburgh, John A. Jansen, Jeroen J. J. P. van den Beucken

**Affiliations:** Department of Biomaterials, Radboudumc, Ph van Leijdenlaan 25, 6525 ex Nijmegen, The Netherlands

**Keywords:** Calcium phosphate, Composite hydrogels, AT-MSCs, BM-MSCs, Osteogenic differentiation

## Abstract

This study aimed to comparatively evaluate the in vitro effect of nanosized hydroxyapatite and collagen (nHA/COL) based composite hydrogels (with different ratios of nHA and COL) on the behavior of human mesenchymal stromal cells (MSCs), isolated from either adipose tissue (AT-MSCs) or bone marrow (BM-MSCs). We hypothesized that (i) nHA/COL composite hydrogels would promote the osteogenic differentiation of MSCs in an nHA concentration dependent manner, and that (ii) AT-MSCs would show higher osteogenic potential compared to BM-MSCs, due to their earlier observed higher proliferation and osteogenic differentiation potential in 2D in vitro cultures [1]. The obtained results indicated that AT-MSCs show indeed high proliferation, differentiation and mineralization capacities in nHA/COL constructs compared to BM-MSCs, but this effect was irrespective of nHA concentration. Based on the results of alkaline phosphatase (ALP) activity and osteocalcin (OCN) protein level, the osteogenic differentiation of BM-MSCs started in the beginning of the culture period and for AT-MSCs at the end of the culture period. At a molecular level, both cell types showed high expression of osteogenic markers (bone morphogenic protein 2 [BMP2], runt-related transcription factor 2 [RUNX2], OCN or COL1) in both an nHA concentration and time dependent manner. In conclusion, AT-MSCs demonstrated higher osteogenic potential in nHA/COL based 3D micro-environments compared to BM-MSCs, in which proliferation and osteogenic differentiation were highly promoted in a time dependent manner, irrespective of nHA amount in the constructs. The fact that AT-MSCs showed high proliferation and mineralization potential is appealing for their application in future pre-clinical research as an alternative cell source for BM-MSCs.

## Introduction

Bone loss, caused by trauma, tumor resection or congenital disorders, is an increasingly serious health problem, for which current treatment remains a clinical challenge, especially for critical size bone defects [2]. Structurally, bone tissue forms the human skeleton, which consists of multiple cell types and a largely mineralized extracellular matrix (ECM). The organic part of ECM is composed of protein fibers (mainly type 1 collagen), which represents about 30 wt% of bone. The inorganic part of bone is composed of minerals (~70 wt% of bone), with hydroxyapatite (HA; Ca_10_(PO_4_)_6_(OH)_2_) as the major component [3, 4]. As such, the weight ratio between collagen and HA is approximately 1:2. These HA crystals, embedded within the extracellular matrix, are very small, measuring approximately 200 Å (in the largest dimension, i.e. nano-sized HA, nHA) [5]. In a synergistic manner, the strands of collagen provide bone with tensile strength and the interspersed nHA crystals provide compressional strength [5].

Nowadays in bone regenerative research, the use of scaffold systems receives remarkable attention, because of the increasing demand to replace autologous bone for grafting purposes. For an appropriate cell attachment, proliferation and osteogenic differentiation, scaffolds need to meet several requirements, e.g. biocompatibility, ability of fluid transport, delivery of bioactive molecules, surface topographical cues, degradability and ability to induce signal transduction [6].

In view of biomimicry, it seems appealing to develop scaffolds that combine structural properties of bone ECM. Among the different materials available for scaffold preparation, hydrogels represent a highly versatile group of biomaterials with appealing properties for 3D scaffolding. Hydrogels are hydrophilic networks (water content ⩾ 30 % by weight) of natural or synthetic polymer chains, which approximate the viscoelastic properties of native tissue [7]. Because of distinctive features, such as biocompatibility, cell-controlled degradability, injectability and ability to release drugs or bioactive molecules, hydrogels are considered as reliable biomaterials for the regeneration of a wide range of tissues, including cartilage and bone [6, 8]. The advantages of natural hydrogels (i.e. collagen, fibrin) include among others their non antigenic and intrinsic cellular interaction capacities [9]. Moreover, collagen based hydrogels support the expression of an osteogenic phenotype of (differentiating) MSCs in vivo [10]. However, the mechanical properties of collagen are relatively low (E ~ 100 MPa) in comparison to bone (E ~ 2-50GPa) [11]. To obtain more biomimetic scaffold systems for bone regeneration, several attempts have focused on the combination of a hydrogel and HA to study effects of cell behavior (mainly mesenchymal stromal cells, MSCs) in vitro and bone regeneration in vivo [12–14]. These studies have shown that HA/collagen based composite hydrogels can have potential to enhance MSC osteogenic differentiation [8, 15].

From a cellular perspective, hydrogels provide a 3D micro-environment to which cells can attach, attain a specific morphology, have 3D cell-cell/cell-biomaterial interactions and subsequently proliferate and differentiate [16]. Moreover, for bone regeneration, the use of cell-based constructs provides osteoinductive properties [17, 18] in comparison to bare scaffolds [19, 20]. Since in the developmental and regenerative processes of bone BM-MSCs are involved, they have become the main cell source for bone tissue engineering [21]. However, MSCs can be extracted from different tissues, such as skin, muscle, periodontal ligament, blood, adipose tissue (AT) and the yield of extracted cells is dependent of cell source. The easiest harvesting of MSCs (less invasive) with substantial yield is from adipose tissue [1, 22]. Moreover, at the same 2D culture condition (using platelet lysate [PL] or fetal bovine serum [FBS] as nutritional supplement), AT-MSCs showed higher proliferation and osteogenic differentiation capacities compared to BM-MSCs, and AT-MCSs showed their highest proliferation and osteogenic differentiation capacities in PL supplemented media, whereas BM-MSCs did in FBS supplemented media [1, 23]. In view of this, the major challenge remains to understand the complexity of cellular responses of different MSCs to different scaffold systems.

The aim of this study was to comparatively evaluate the in vitro effect of biomimetic nHA/collagen based composite hydrogels (with different ratios of nHA) on the behavior of human MSCs, isolated from adipose tissue (AT-MSCs) or bone marrow (BM-MSCs). We hypothesized that (i) nHA/collagen based hydrogels will promote the osteogenic differentiation of MSCs in an nHA concentration dependent manner, (ii) AT-MSCs will show higher osteogenic potential compared to BM-MSCs, because of their intrinsic higher proliferation and osteogenic differentiation potential in 2D in vitro cultures.

## Materials and Methods

### Cell Culture

AT-MSCs were isolated from fat tissue of healthy human donors. The fat tissue was obtained from the Department of Plastic Surgery (Radboudumc, The Netherlands) after written informed consent. BM-MSCs were isolated from human iliac bone chips, obtained from patients undergoing maxillofacial surgery at the Department of Oral and Craniofacial Surgery (Radboudumc, The Netherlands) after written informed consent. MSC extraction was performed according to the principles of the Declaration of Helsinki**.** The isolation procedure of AT-MSCs and BM-MSCs is described in detail elsewhere [1, 24–26]. Harvested cells were examined by fluorescence-activated cell sorting (FACS) for positive expression of CD73, CD90 and CD105 (eBioscience, San Diego, USA) and negative expression of CD45 (R&D system, Abingdon, United Kingdom). Next, both cell types were examined (via biochemical assays) for their osteogenic potential, i.e. ALP activity and calcium deposition.

Upon usage, MSCs were cultured in corresponding proliferation media consisting of alpha Minimal Essential Medium, (α-MEM; Gibco®, Life Technologies, Grand Island, USA) supplemented either with 5 % PL (Sanquin Blood Bank, The Netherlands) for AT-MSCs or with 15 % fetal bovine serum (FBS; Lonza, Basel, Switzerland) for BM-MSCs [1], at 37°C in humid atmosphere with 5 % CO_2_ (the complete composition of proliferation media is given in Table 1). Medium was changed twice a week. Cells were passaged upon reaching ~80 % confluency using 0.25%*w*/*v* trypsin/0.02 % EDTA (Gibco®).

**Table 1:** Composition of the proliferation media (PM) and osteogenic media (OM)

BM-MSCs	AT-MSCs
Minimal Essential Medium (α-MEM)	Minimal Essential Medium (α-MEM)
FBS-supplemented (PM-FBS)	PL-Supplemented (PM-PL)
15 % fetal bovin serum (FBS)	5 % platelet lysate (PL)
0.2 mM L-ascorbic acide 2-phosphate (Vit C)	10 U/ml heparin
2 mM L-glutamine	100 U/ml penicillin
100 U/ml penicillin	10 μg/ml streptomycin
10 μg/ml streptomycin	
FBS-supplemented (OM-FBS)	PL-Supplemented (OM-PL)
15 % fetal bovin serum (FBS)	5 % platelet lysate (PL)
0.2 mM L-ascorbic acide 2-phosphate (Vit C)	0.2 mM L-ascorbic acide 2-phosphate (Vit C)
2 mM L-glutamine	2 mM L-glutamine
100 U/ml penicillin	100 U/ml penicillin
10 μg/ml streptomycin	10 μg/ml streptomycin
10–8 M dexamethasone	10–8 M dexamethasone
0.01 M β-glycerophosphate	0.01 M β-glycerophosphate
	0.02 10 U/ml heparin

### Preparation of Hydrogels and Experimental Groups

Prior to the preparation of hydrogel scaffolds, nHA crystals (size: 20–500 nm; Berkeley Advanced Biomaterials, Berkeley, CA, USA) were suspended in PBS (10× concentrated) at a final concentration of 150 mg/ml. The suspension was homogenized by sonication for 20 min. Before addition to hydrogels (see Table 2), this suspension was vortexed for 1 min. For the preparation of hydrogels, collagen type 1 (COL; rat tail; BD Bioscience, Bedford MA, USA) was used with various amounts of nHA (Table 2). The procedure of hydrogel preparation was according to the manufacturer’s instruction (Table 2), and composite nHA/COL hydrogels were prepared with an nHA/COL ratio (wt/wt) of 0/1, 1/1, and 2/1. MSCs were added during hydrogel preparation (Table 2). Cell seeding density of AT-MSCs and BM-MSCs in all experimental groups was 1x10^6^ per 1 ml of hydrogels (Table 3).

**Table 2:** Reagents for scaffold preparation and cell encapsulation

Groups	A. Without cells		B. With the cells	
CaP/Collagen 0:1 (control)	Reagents	Volume (μL)	Reagents	Volume (μL)
	Collagen	2610	Collagen	2610
	PBS(10×)	300	PBS(10×)	300
	CaP susp.	-	CaP susp.	-
	NaOH 1 N	60	NaOH 1 N	60
	H_2_O/α-MEM	30	H_2_O/α-MEM	0
	Cell susp.	–	Cell susp.	30
	Total	3000 μl	Total	3000 μl
CaP/Collagen 1:1	Reagents	Volume (μL)	Reagents	Volume (μL)
	Collagen	2610	Collagen	2610
	PBS(10×)	240	PBS(10×)	240
	CaP susp.	60(150 mg/ml)	CaP susp.	60(150 mg/ml)
	NaOH 1 N	60	NaOH 1 N	60
	H_2_O/α-MEM	30	H_2_O/α-MEM	0
	Cell susp.	–	Cell susp.	30
	Total	3000 μl	Total	3000 μl
CaP/Collagen 2:1	Reagents	Volume (μL)	Reagents	Volume (μL)
	Collagen	2610	Collagen	2610
	PBS(10×)	180	PBS(10×)	180
	CaP susp.	120(150 mg/ml)	CaP susp.	120(150 mg/ml)
	NaOH 1 N	60	NaOH 1 N	60
	H_2_O/α-MEM	30	H_2_O/α-MEM	
	Cell susp.	–	Cell susp.	30
	Total	3000 μl	Total	3000 μl

**Table 3:**
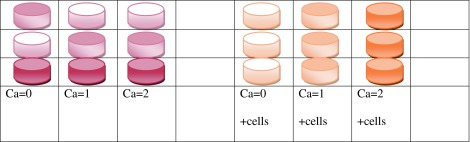
Schematic overview of the experimental groups used with varying CaP-particle content (Ca) and with/- cells

For the analysis of cellular behavior (DNA content, ALP activity and calcium [Ca] deposition) and histological analysis (HE staining, Von Kossa staining and immunohistochemistry [IHC]) hydrogels were injected in 48 well plates, with the total hydrogel volume of 200 μl (200.000 cells; *n* = 3). To obtain sufficient RNA, hydrogels for RNA extraction were injected in 24 well plates, with the total volume of 400 μl (400.000 cells; *n* = 3). All samples were incubated in corresponding osteogenic media (Table 1), supplemented with either 5 % PL for AT-MSCs or 15 % FBS for BM-MSCs and incubated for 35 days at 37 °C in a humid atmosphere with 5 % CO_2_. To monitor the behavior of pure hydrogels (without cells) as a negative control nHA/COL = 0/1, nHA/COL = 1/1, nHA/COL = 2/1 constructs were prepared and cultured either in PL or in FBS supplemented media. Cell morphology was monitored with an inverted light microscope (Leica DM-IL, 5 W LED illumination, Rijswijk, The Netherlands).

### Cell Behavior

To monitor cellular behavior, cellular DNA content, alkaline phosphatase (ALP) activity and calcium deposition were analyzed [1]. Samples were collected (at days 1, 14, 28 and 35) in 1 ml MilliQ and subsequently stored at −80 °C until use. The same samples were used for all biochemical assays. For extraction of cells from hydrogels, scaffolds were digested first using Collagenase A (Roche Diagnostics Penzberg, Germany). According to the manufacturer’s instruction, a digestion buffer was prepared in a concentration of 1 mg/ml. Before digestion, the constructs were washed 2 times with PBS, incubated with 1 ml digestion buffer overnight at 37 °C on a rotary shaker. After complete digestion and two repetitive freeze/thaw cycles at -80 °C/+20 °C, samples were ready for the analysis of DNA content, ALP activity and Ca deposition [1, 27]. The results of ALP content were normalized by the corresponding cellular dsDNA amount. After analyzing DNA content and ALP activity, samples were doubled in volume using 1 N acetic acid (to dissolve mineral deposits) and incubated at room temperature overnight. Ca deposition was measured as described previously [28].

#### Cellular DNA Content

Cellular DNA content was measured using a QuantiFluor® dsDNA System Kit (Promega, Madison, Wisconsin, USA). For the standard curve, serial dilutions of dsDNA stock (range: 0–2000 ng/ml) were prepared. 100 μl of either sample of standard solution was added into the wells, followed by 100 μl of working solution. The plate was incubated at room temperature for 5 min, and then the absorbance of samples/standards was measured at 504 nm excitation and 541 nm emission, using a fluorescence microplate reader (FL600, BioTek, Canada).

#### Alkaline Phosphatase (ALP) Activity

ALP activity was measured using a 5 nM p-nitrophenyl phosphate (4-NP) colorimetric assay. The procedure was according to the manufacturer’s instruction (Sigma-Aldrich, St. Louis, MO, USA). Briefly, 80 μl of sample solution was combined with 20 μl of buffer (0.5 M 2-amino-2methyl-1-propanol). A standard curve was prepared with serial dilutions of 4-NP, in a range of 0–25 nmol. Next, 100 μl substrate solution (5 nM p-nitrophenyl phosphate) was added to the samples/standards and incubated for 60 min at 37°C. The reaction was stopped by adding to each well 50 μl 0.3 M NaOH and the absorbance of samples was measured at 405 nm using an ELISA microplate reader (EL800, BioTek, Abcoude, The Netherlands). ALP activity was normalized for corresponding cellular dsDNA amount.

#### Calcium Deposition

Calcium deposition was measured using the orthocresolphtalein complexone assay (OCPC; Sigma Aldrich, St. Louis, MO, USA), which is based on a colorimetric reaction between o-cresolphthalein complexone and calcium. The assay was performed according to the manufacturer’s instruction. For the standard curve, serial dilutions of calcium stock (CaCl_2_) were prepared (range: 0–100 mg/ml). Next, 10 μl of sample or standard was used, to which 300 μl OCPC solution was added to complete the reaction. After the incubation of the plate for 10 min at room temperature, the absorbance was measured at 570 nm using an ELISA microplate reader (EL800, BioTek, Canada).

#### RNA Isolation and Reverse Transcription

To analyze gene expression profiles of selected genes, cellular RNA was isolated using Tryzol method in combination with the Genelute Mammalian Total RNA Miniprep Kit (Sigma-Aldrich). Samples were collected (at days 0, 14, 28 and 35) in 1 ml TRIzol lysis buffer and subsequently stored at -80 °C until RNA extraction. After extraction, RNA was quantified using a spectrophotometer (Nanodrop Technologies, Wilmington, DE, USA). For reverse transcription (cDNA synthesis), an iScript™ cDNA kit was used (Bio-Rad, Hercules, CA, USA), and for each cDNA reaction 1 μg of RNA was used. The samples were stored at -20 °C until use.

#### Real-Time Polymerase Chain Reaction

For real-time polymerase chain reaction (RT-PCR), qPCR Master Mix Plus/SYBR Green I (Eurogentec; Seraing, Belgium) was used. RT-PCR was completed with 40 amplification cycles. The sequence of applied primers is given in Table 4. The raw data were normalized to the expression of Ribosomal Protein Large P0 (RPLP0 housekeeping gene) within the same sample/RNA [29]. Gene expression level and fold changes were calculated according to Livak & Schmittgen (2^-ΔΔCt^) relative subsequently to AT-MSCs or BM-MSCs at the day 0 [30]. The genes of interest were Osteocalcin (OCN), Bone Morphogenetic Protein 2 (BMP2), Runt-related Transcription Factor 2 (RUNX2), and Collagen type 1 (COL1).

**Table 4:** Primer sequences for RT-PCR

Gene name	Sequences
RPLP0	Forward-TTCTTCTTTGGGCTG GTCAT
	Reverse-TTGGGTAGCCAATCTGCAGA
RUNX2	Forward-TCTGGCCTTCCACTCTCAGT
	Reverse-GACTGGCGGGGTGTAAGTAA
BMP-2	Forward-CCCAGCGTGAAAAGAGAGAC
	Reverse-GGAAGCAGCAACGCTAGAAG
COL1	Forward-GGTGTAAGCGGTGGTGGTTAT
	Reverse-AGGTTCCCCGTTCTCACTTT
OCN	Forward- GGCGCTACCTGTATCAATGG
	Reverse-GTGGTCAGCCAACTCGTCA

## Histological Analysis and Immunohistochemistry

Samples for histological analysis and IHC were collected at day 7 and 28, fixed at 10 % formalin, decalcified in 4 % EDTA for ~2 weeks, and regularly checked with X-ray for the level of remaining mineral content in the constructs. After complete demineralization, samples were dehydrated in a graded series of ethanol (70–100 %) and embedded in paraffin. Simultaneously, human bone chips (obtained from the Department of Maxillofacial surgery, Radboudumc, Nijmegen, The Netherlands; after written informed consent), were processed as a control for all stains. Sections with a thickness of 6 μm were prepared using a standard microtome (RM2165; Leica, Nussloch, Germany). To analyze cell distribution in hydrogels, every 10th slide was stained with hematoxylin/eosin (HE). To identify phosphate groups in mineralized matrix, separate slides were prepared for Von Kossa staining, which were stained first with 5 % silver nitrate (AgNO_3_), washed with distilled water, dehydrated again, and fixed with 5 % sodium thiosulfate (Na_2_S_2_O_3_).

Subsequently, continuous tissue sections were used to monitor osteogenic differentiation of cells in hydrogels. As an osteogenic marker, osteocalcin (OCN, rabbit anti-mouse osteocalcin) protein was cheeked by IHC. Sections were de-paraffinised, rehydrated and rinsed in PBS. Next, samples were fixed for 10 min in 10 % hydrogen peroxide (stock)/methanol solution. Afterwards, samples were pre-incubated for 10 min with 10 % normal donkey serum (NDS) and then incubated with the primary antibody (1:1600) overnight at 4 °C. Subsequently, sections were washed 3× with PBS, and incubated with secondary antibody, anti mouse IgG (host donkey) conjugated with biotin (Jackson ImmunoResearch, West Baltimore Pike, West Grove, PA, USA) for 60 min. After washing, the peroxidase conjugates were visualized with 3′3diaminobenzidine (DAB) substrate (Envision kit; Dako Cytomation) for 10 min at room temperature, and nuclei were stained for 10 s with hematoxilin.

## Statistical Analysis

Data are presented as mean ± standard deviation. Statistical analysis was performed based on N = 3 (for all experimental groups) with Graphpad Prism® 5.03 software (Graphpad Software Inc., San Diego, CA, USA). Quantitative results were analyzed using a one-way ANOVA with a Posthoc Dunnett test (using either nHA/COL = 0/1 or day 7 [for biochemical assays] and day 14 [for Q-PCR analysis] data as control). Differences were considered significant at *p* < 0.05. All experiments were repeated 4 times.

## Results

The results of FACS analysis showed that both cell types (AT-MSCs and BM-MSCs) were 99 % positive for expression of stem cell surface markers CD73, CD90 and CD105, whereas both these cell types were completely negative for the hematopoietic marker CD45 (data not shown).

### Cell Morphology

**Fig. 1:**
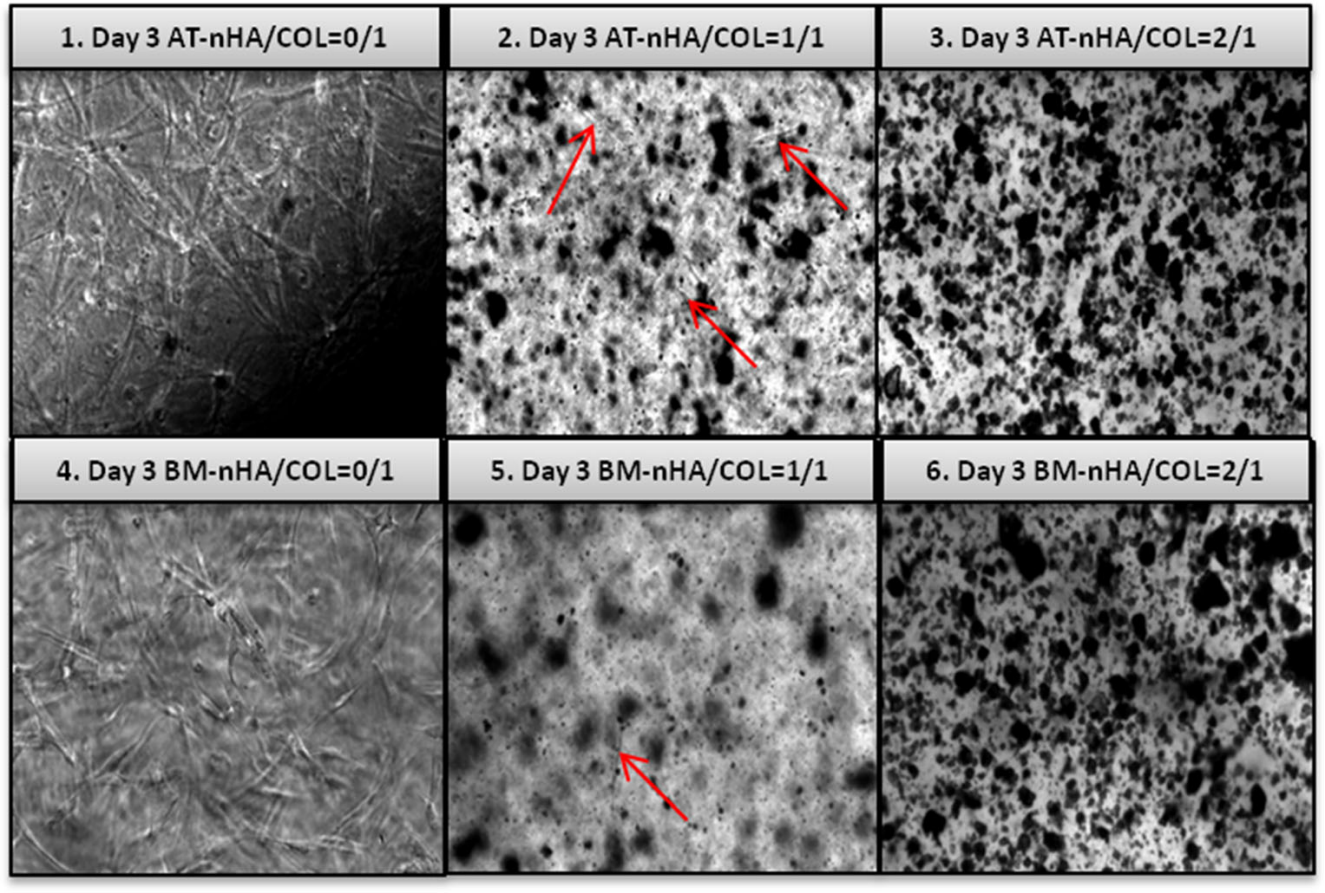
Cell morphology of AT-MSCs or BM-MSCs monitored with inverted light microscopy in different nHA/COL constructs, after 3 days of culture

Light microscopy analysis showed that both AT-MSCs and BM-MSCs were able to survive in nHA/COL constructs (Fig. 1). Both cell types showed an elongated, spindle-shaped morphology in these constructs, but from 3 days of culture onward, AT-MSCs visually appeared at higher cell density than BM-MSCs. After 7 days, hydrogels changed their shape and morphology, becoming dense and no longer transparent for light microscopy evaluation. The constructs shrunk drastically by changing diameter size about 2-fold (from ~11 mm to ~4-5 mm).

### Cellular Behavior

#### Cellular DNA Content

**Fig. 2:**
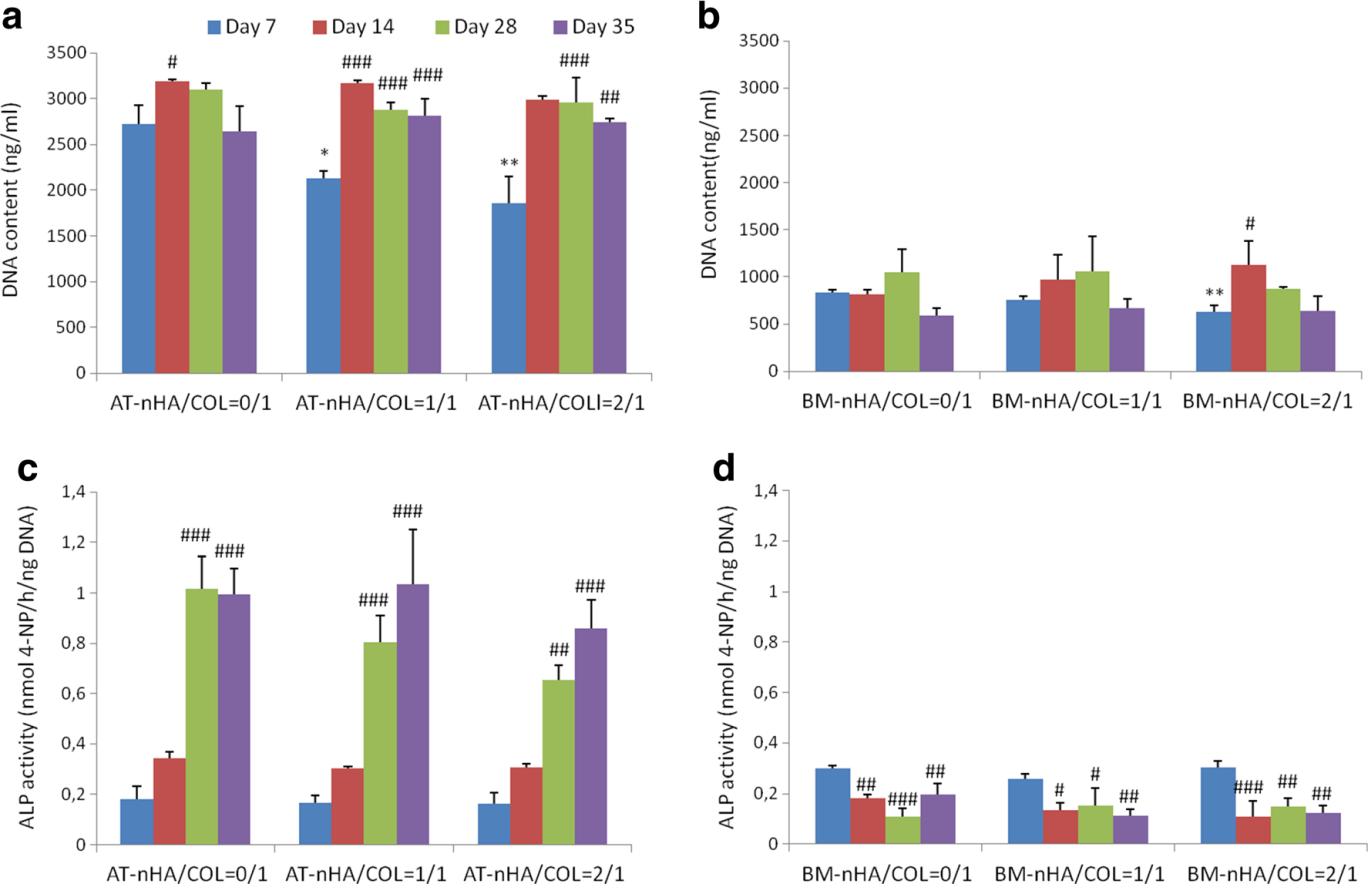
Cellular behavior of MSCs in different nHA/COL constructs. **a** Cellular DNA content of AT-MSCs, **b** Cellular DNA content BM-MSCs **c** ALP-activity of AT-MSCs, (D) ALP-activity of BM-MSCs. The “*” indicates significantly different compared to nHA/COL = 0/1 control, at the same time point (*p*-values: *p < 0.05, **p < 0.01, ***p < 0.001), “^#^” indicates significantly different compared to day 7 (*p*-values: ^#^ *p* < 0.05, ^##^ *p* < 0.01, ^###^ *p* < 0.001)

AT-MSCs showed a gradual increase in cell proliferation until day 14 (Fig. 2a). A significant temporal increase in cellular DNA content (relative to day 7) was observed for AT-nHA/COL = 0/1 at day 14 (*p* < 0.05), AT-nHA/COL = 1/1 at day 14, 28, 35 (*p* < 0.001), and AT-nHA/COL = 2/1 at day 14, (*p* < 0.001). Relative to AT-nHA/COL = 0/1, at day 7, a significantly lower cellular DNA content was observed for AT-nHA/COL = 1/1 (*p* < 0.05) and AT-nHA/COL = 2/1 (*p* < 0.01).

BM-MSCs (Fig. 2b), showed limited cell proliferation over the entire culture period. Relative to day 7, a significant increase in cellular DNA content was observed only for BM-nHA/COL = 2/1 at day 14 (*p* < 0.05).

#### ALP Activity

AT-MSCs showed a gradual increase in ALP-activity (early marker for osteogenic differentiation) until the end of the culture period, irrespective of construct type (Fig. 2c). A significant increase in ALP-activity (relative to day 7) was observed for AT-nHA/COL = 0/1 and AT-nHA/COL = 1/1 at day 28 and 35 (*p* < 0.001), and AT-nHA/COL = 2/1 at days 28 (*p* < 0.01) and 35 (*p* < 0.001). Relative to AT-nHA/COL = 0/1, no significant differences were observed for AT-nHA/COL = 1/1 and AT-nHA/COL = 2/1.

In contrast to AT-MSCs, very low ALP-activity was observed for BM-MSCs (Fig. 2d) over the entire culture period, which was significantly decreased (relative to day 7) until the end of culture in all experimental constructs. Relative to BM-nHA/COL = 0/1, no significant differences were found between different constructs.

#### Calcium Deposition

**Fig. 3:**
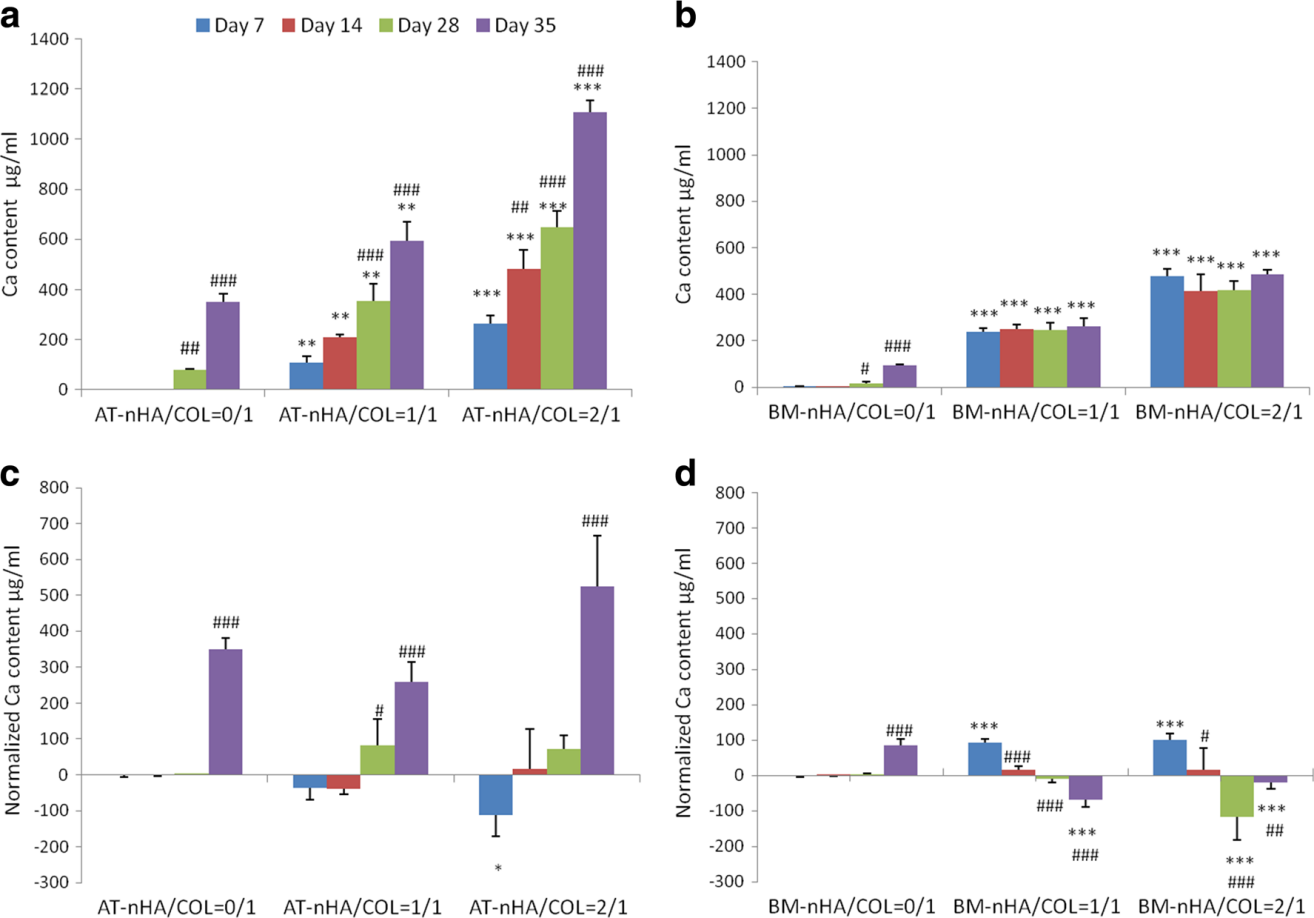
Ca deposition of MSCs in different nHA/COL constructs. **a** Ca deposition of AT-MSCs, **b** Ca deposition of BM-MSCs **c** Normailzed Ca deposition of AT-MSCs, **d** Normailzed Ca deposition of BM-MSCs. The “*” indicates significantly different compared to nHA/COL = 0/1 control, at the same time point (*p*-values: *p < 0.05, **p < 0.01, ***p < 0.001), “^#^” indicates significantly different compared to day 7 (*p*-values: ^#^ *p* < 0.05, ^##^ *p* < 0.01, ^###^ *p* < 0.001)

Calcium deposition results (Fig. 3a-b) were normalized for non-cellular constructs, which were treated similarly as cellular constructs. The mineralization level for both cell types was generally low. Relative to day 7, AT-MSCs showed a temporal increase in calcium deposition (Fig. 3c) for AT-nHA/COL = 1/1 at day 28 (*p* < 0.05), and at day 35 (*p* < 0.001) for all experimental constructs.

BM-MSCs generally showed a decrease in mineralization (relative to day 7) starting at day 14 until the end of the culture period (Fig. 3d). Only for BM-HA/COL = 0/1, an increase in mineralization was observed at day 35 (*p* < 0.001). Relative to BM-nHA/COL = 0/1, a significantly increased mineralization was observed for BM-nHA/COL = 1/1 and BM-nHA/COL = 2/1 (*p* < 0.001) at day 7.

#### Gene Expression

##### BMP2

**Fig. 4:**
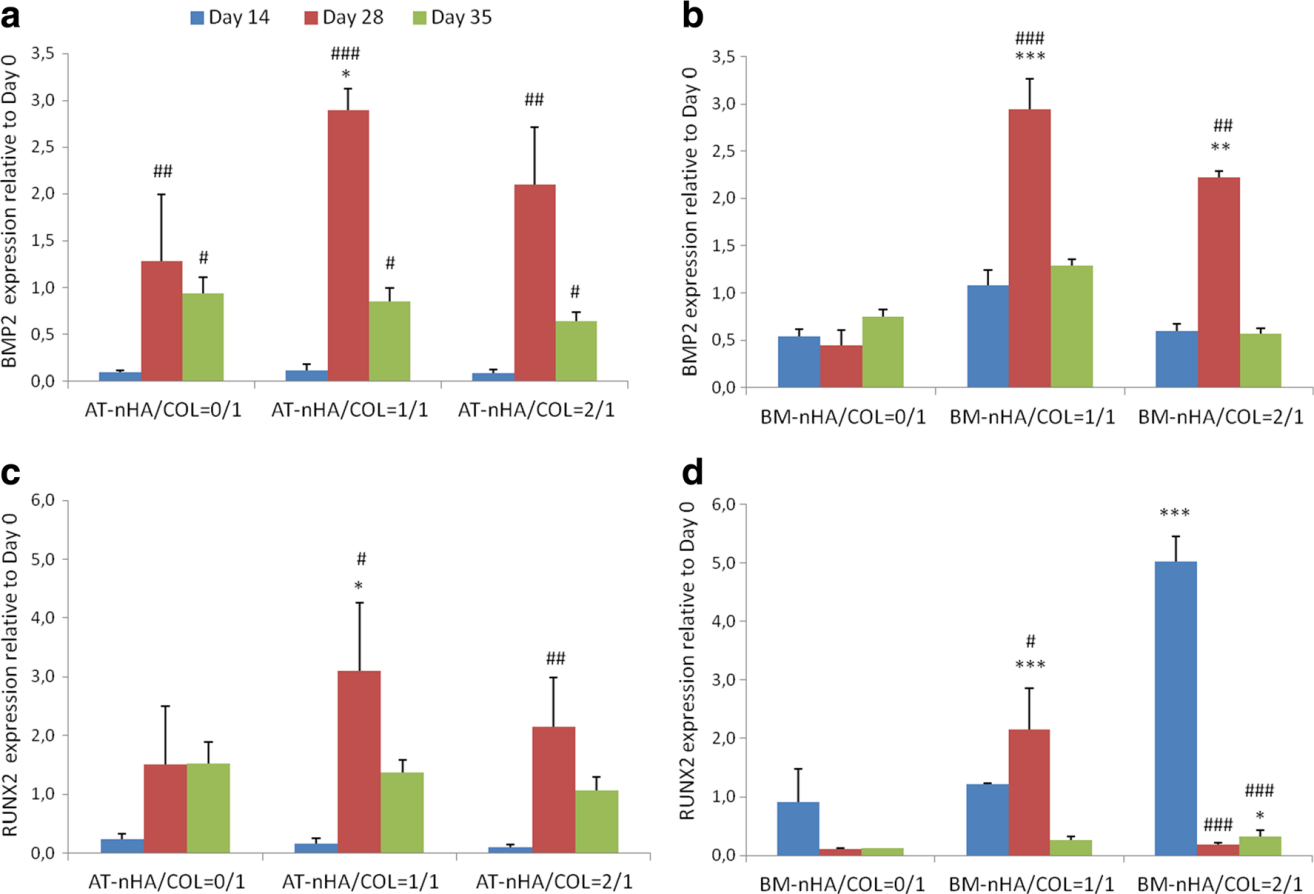
Gene expression profiles of BMP2 and RUNX2. **a** Gene expression of BMP2 in AT-MSCs, **b** Gene expression of BMP2 in BM-MSCs, **c** Gene expression of RUNX2 in AT-MSCs, **d** Gene expression of RUNX2 in BM-MSCs. The “*” indicates significantly different compared to nHA/COL = 0/1 control, at the same time point (*p*-values: *p < 0.05, **p < 0.01, ***p < 0.001), “^#^” indicates significantly different compared to day 14 (*p*-values: ^#^ *p* < 0.05, ^##^ *p* < 0.01, ^###^ *p* < 0.001)

AT-MSCs showed a gradual increase in BMP2 expression until day 28, and decrease again until day 35, irrespective of construct type (Fig. 4a). Relative to day 14, significantly higher BMP2 expression was observed for AT-nHA/COL = 0/1 at day 28 (*p* < 0.01) and 35 (*p* < 0.05), for AT-nHA/COL = 1/1 at day 28 (*p* < 0.001) and 35 (*p* < 0.05), and for AT-nHA/COL = 2/1 at day 28 (*p* < 0.01) and 35 (*p* < 0.05). Relative to AT-nHA/COL = 0/1, significantly higher BMP2 expression was observed for AT-nHA/COL = 1/1 at day 28 (*p* < 0.05).

BM-MSCs showed a gradual increase in BMP2 expression until day 28 and thereafter a decrease until day 35 (Fig. 4b) for BM-nHA/COL = 1/1 and BM-nHA/COL = 2/1. Relative to day 14, significantly higher BMP2 expression was observed for BM-nHA/COL = 1/1 and for BM-nHA/COL = 2/1 at day 28 (*p* < 0.001). Relative to BM-nHA/COL = 0/1, significantly higher BMP2 expression was observed for BM-nHA/COL = 1/1 and for BM-nHA/COL = 2/1 at day 28 (*p* < 0.01).

##### RUNX2

AT-MSCs showed an upregulation of RUNX2 expression after day 14, irrespective of construct type (Fig. 4c). Relative to day 14, significantly higher RUNX2 expression was observed for AT-nHA/COL = 1/1 at day 28 (*p* < 0.05) and for AT-nHA/COL = 2/1 at day 28 (*p* < 0.01).

BM-MSCs, showed high levels of RUNX2 expression at day 14 irrespective to construct type, which further increased only for BM-nHA/COL = 1/1 (Fig. 4d). Relative to day 14, significantly higher RUNX2 expression was observed for BM-nHA/COL = 1/1 at day 28 (*p* < 0.05). Relative to BM-nHA/COL = 0/1 significantly higher RUNX2 expression was observed for BM-nHA/COL = 1/1 at day 28 (*p* < 0.001) and for BM-nHA/COL = 2/1 at day 14 (*p* < 0.001).

##### Ocn

**Fig. 5:**
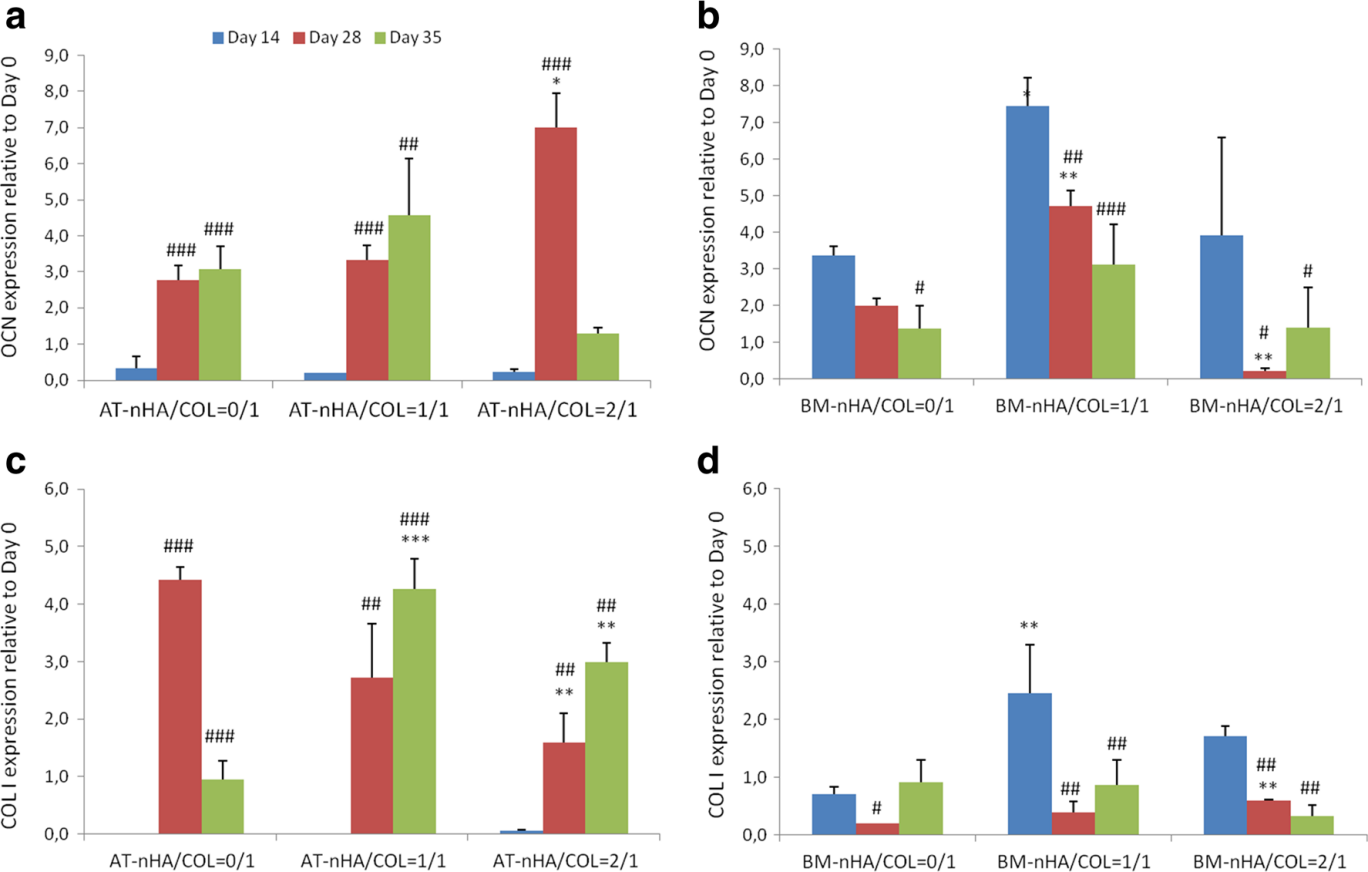
Gene expression profiles of OCN and COL1. **a** Gene expression of OCN in AT-MSCs, **b** Gene expression of OCN in BM-MSCs, **c** Gene expression of COL1 in AT-MSCs, **d** Gene expression of COL1 in BM-MSCs. The “*” indicates significantly different compared to nHA/COL = 0/1 control, at the same time point (*p*-values: *p < 0.05, **p < 0.01, ***p < 0.001), “^#^” indicates significantly different compared to day 14 (*p*-values: ^#^ *p* < 0.05, ^##^ *p* < 0.01, ^###^ *p* < 0.001)

AT-MSCs showed an upregulation of OCN expression starting at day 28 until day 35, except for AT-nHA/COL = 2/1, for which the expression of OCN was downregulated after day 28 (Fig. 5a). Relative to day 14, significantly higher OCN expression was observed for AT-nHA/COL = 0/1 at day 28 and 35 (*p* < 0.001), for AT-nHA/COL = 1/1 at day 28 (*p* < 0.001) and 35 (*p* < 0.01), and for AT-nHA/COL = 2/1 at day 28 (*p* < 0.001). Relative to AT-nHA/COL = 0/1, significantly higher OCN expression was observed for AT-nHA/COL = 2/1 at day 35 (*p* < 0.05).

BM-MSCs, showed high expression of OCN at day 14, which was downregulated toward the end of the culture period, irrespective of construct type (Fig. 5b). Relative to day 14, significantly lower OCN expression was observed for BM-nHA/COL = 0/1 at day 35 (*p* < 0.05), for BM-nHA/COL = 1/1 at day 28 (*p* < 0.01) and 35 (*p* < 0.001) and for BM-nHA/COL = 2/1 at day 28 and 35 (*p* < 0.05). Relative to BM-nHA/COL = 0/1, an upregulation of OCN expression was observed for BM-nHA/COL = 1/1 at day 14 (*p* < 0.05) and 28 (*p* < 0.01).

##### COL1

AT-MSCs showed an upregulation of COL1 expression starting at day 28 (Fig. 5c), irrespective of construct type. Significantly higher COL1 expression (relative to day 14) was observed for AT-nHA/COL = 0/1 at day 28 and 35 (*p* < 0.001), for AT-nHA/COL = 1/1 at day 28 (*p* < 0.01) and 35 (*p* < 0.001), and for AT-nHA/COL = 2/1 at day 28 (*p* < 0.01) and 35 (*p* < 0.01). Relative to AT-nHA/COL = 0/1, the expression of COL1 was significantly higher for AT-nHA/COL = 1/1 at day 35 (*p* < 0.001) and for AT-nHA/COL = 2/1 at day 28 and 35 (*p* < 0.01).

BM-MSCs showed high expression of COL1 at day 14, after which the COL1 expression was downregulated until the end of the culture period (Fig. 5d). Relative to day 14, significantly lower COL1 expression was observed for BM-HA/COL = 0/1 at day 28 (*p* < 0.05), for BM-HA/COL = 1/1 at day 28 and 35 (*p* < 0.01) and for BM-HA/COL = 2/1 at day 28 and 35 (*p* < 0.01). Relative to BM-HA/COL = 0/1, significantly higher COL1 expression was for BM-HA/COL = 1/1 at day 14 (*p* < 0.01) and for BM-HA/COL = 2/1 at day 28 (*p* < 0.01).

#### Histological Analysis and Immunohistochemistry

##### HE Stain

**Fig. 6:**
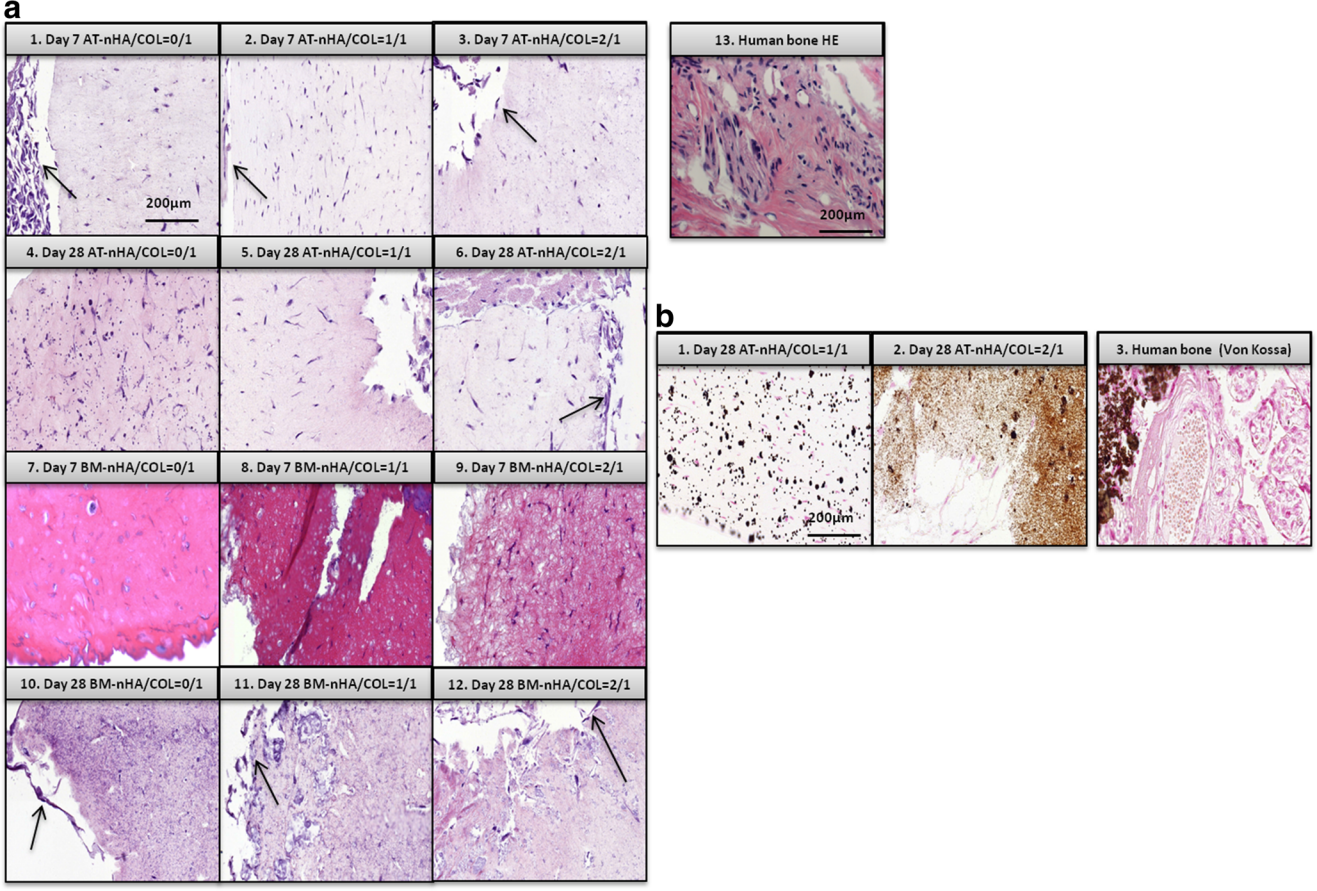
**a** HA staining of different group of nHA/COL constructs after 7 and 28 days of culture, **b** Von Kossa staining of different group of nHA/COL constructs after 28 days of culture

**Fig. 7:**
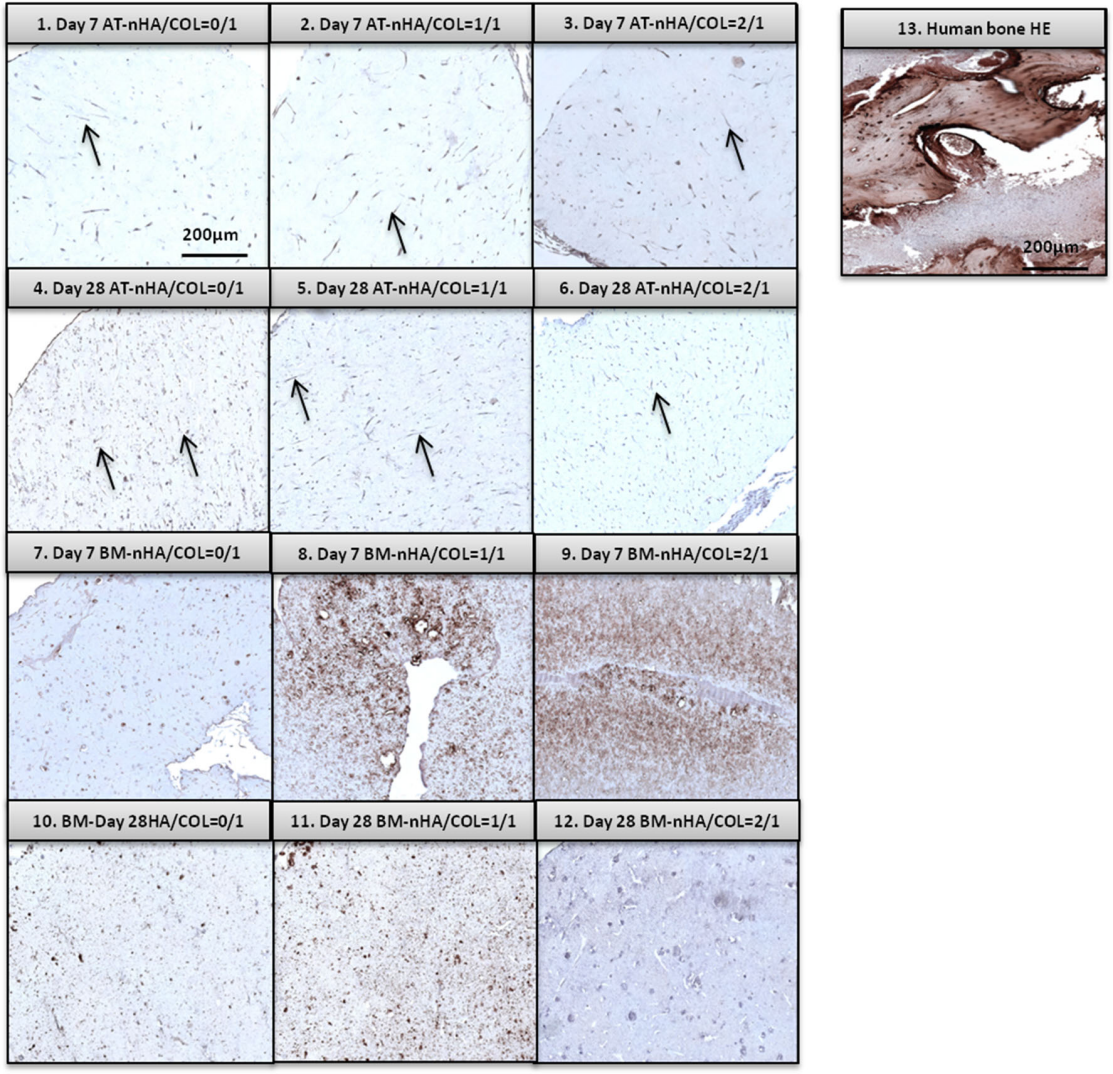
Immunohistochemical staining of OCN, in different group of nHA/COL constructs, after 7 and 28 days of culture

HE-stained histological sections of all experimental constructs as well as HE stained sections of human bone (positive control) are presented in Fig. 6a. Results of AT-MSC constructs showed that at day 7 as well as at day 28, cells were distributed throughout the entire construct. At the periphery of constructs, the cellular density was apparently higher compared to the centre of constructs (high density cell populations are indicated with arrows in Fig. 6a-1, 2, 3, 5). The distribution of BM-MSCs in nHA/COL constructs was different at different time points. At day 7, cells were homogeneously distributed throughout the entire construct (Fig. 6a-7, 8, 9). However, at day 28 cells were mainly located at the periphery of constructs, and only few cells were detectable in the central region (Fig. 6a-10, 11, 12).

##### Von Kossa Stain

To monitor the mineral deposition inside the constructs, Von Kossa staining was used (Fig. 6b). Mineral deposition was only detectable for AT-nHA/COL = 1/1 and AT-nHA/COL = 2/1 at day 28.

##### IHC-OCN Stain

AT-MSCs showed detectable OCN expression at day 28, especially for AT-nHA/COL = 0/1 (Fig. 7-4, 5, 6). OCN protein was homogeneously distributed throughout the entire construct. AT-MSCs were observed in these constructs with an elongated, spindle-shaped morphology (highlighted with arrows in Fig. 7-4, 5, 6). BM-MSCs showed high OCN expression at day 7, especially for BM-nHA/COL = 1/1 and for BM-nHA/COL = 2/1 (Figs. 7-8, 9). At day 28, the OCN was still detectable for BM-nHA/COL = 0/1 and BM-nHA/COL = 1/1, but not for BM-nHA/COL = 2/1 (Figs. 7–10, 11, 12).

## Discussion & Conclusion

The aim of this study was to comparatively evaluate the in vitro effect of biomimetic nHA/collagen based composite hydrogels (with different concentrations of nHA) on the behavior of MSCs, isolated from adipose tissue (AT-MSCs) and bone marrow (BM-MSCs). We hypothesized that (i) nHA/collagen based hydrogels promote the osteogenic differentiation of MSCs in nHA concentration dependent manner, and that (ii) AT-MSCs will show higher osteogenic capacities compared to BM-MSCs, because of their intrinsic proliferation and osteogenic differentiation potential in 2D in vitro cultures. This study indicated that AT-MSCs show high proliferation, differentiation and mineralization potential in nHA/COL constructs, irrespective of nHA concentration, whereas BM-MSCs showed only marginal cell proliferation and osteogenic differentiation in all experimental nHA/COL constructs. Based on the results of ALP activity and OCN protein level, the osteogenic differentiation of BM-MSCs started in the beginning of the culture period and for AT-MSCs at the end of the culture period. At a molecular level, both cell types showed high expression of osteogenic markers (BMP2, RUNX2, OCN or COL1) in both a nHA concentration and time dependent manner.

At a cellular level, the material properties (different nHA/COL ratio) did not show any substantial effect on the cellular behavior of AT-MSCs or BM-MSCs. However, apparent differences were observed between cell types, i.e. the source for MSCs. AT-MSCs showed higher proliferation and osteogenic differentiation compared to BM-MSCs. This phenomenon corroborates several earlier studies [1, 23], but this study clearly demonstrates that the cellular behavior of AT-MSCs does not change in a nHA concentration dependent manner. High proliferation levels of AT-MSCs could potentially provide faster cell-cell interaction compared to BM-MSCs, and this cellular interaction in 3D micro-environment could promote subsequent AT-MSC osteogenic differentiation [23, 31]. Remarkably, BM-MSCs started to proliferate and differentiate already within the first week of culture, irrespective of nHA concentration, whereas AT-MSCs started to differentiate only after the third week of culture. Early osteogenic differentiation of BM-MSCs can be explained with faster osteocyte formation. The signaling mechanisms involved in osteocyte formation could be activated at different time points in AT-MSCs and BM-MSCs, because AT-MSCs and BM-MSCs were cultured in different (PL or FBS supplemented) osteogenic media with different content of signaling molecules [32].

Molecular analysis of osteogenic markers, i.e. BMP2 (osteoblast differentiation marker), RUNX2 (essential element for osteogenic differentiation and skeletal morphogenesis), OCN (later stage osteogenic differentiation marker) and COL1 (crucial element of connective tissue) [33–35] showed that the material properties (nHA concentration) have an effect on the gene expression pattern of these molecules. It is known that nHA play a functional role in integrin-mediated cell adhesion and signaling [36]. Cell adhesion to ECM is mediated by transmembrane receptors [37]. This initiates intracellular signals, and focal adhesion kinase activation [38], which play a crucial role in activation of downstream signaling. This signaling cascade can stimulate the activation of transcription factors and subsequent signal transduction [39]. For AT-MSCs, the expression of BMP2, RUNX2 and COL1 was promoted by AT-nHA/COL = 1/1 constructs, whereas the expression of OCN and COL1 was promoted by AT-nHA/COL = 2/1 constructs.

For BM-MSCs the expression pattern of BMP2, RUNX2, OCN and COL1 was remarkably higher in BM-nHA/COL = 1/1 constructs, especially in the beginning of the culture period (at day 14). This observation proved that the osteogenic differentiation of BM-MSCs starts at an earlier time points compared to AT-MSCs. Moreover, already at day 7, BM-MSCs expressed high levels of OCN, which also indicates early osteogenic differentiation of BM-MSCs. Next, AT-MSCs and BM-MSCs showed a similar pattern of BMP2 expression. It has been shown that AT-MSCs can respond to BMP2 signals and express other osteogenic markers (e.g. OPN and RUNX2) [40], which indicates that AT-MSCs are a reliable cell type for future use in cell-based bone regenerative strategies.

In conclusion, AT-MSCs demonstrated higher osteogenic potential in nHA/COL based 3D micro-environments compared to BM-MSCs, in which proliferation and osteogenic differentiation were highly promoted. The proliferation and osteogenic differentiation pattern of AT-MSCs and BM-MSCs was regulated in a time dependent manner, irrespective of nHA amount in the constructs. On the other hand, nHA/COL ratios differently affected gene expression profiles of AT-MSCs and BM-MSCs. The fact that AT-MSCs showed high proliferation and mineralization is appealing for their application in future (pre-)clinical research as an alternative cell source for MSCs.
